# Down-regulation of a pro-apoptotic pathway regulated by PCAF/ADA3 in early stage gastric cancer

**DOI:** 10.1038/s41419-018-0470-8

**Published:** 2018-04-18

**Authors:** Daniella Brasacchio, Rita A. Busuttil, Tahereh Noori, Ricky W. Johnstone, Alex Boussioutas, Joseph A. Trapani

**Affiliations:** 1 Sir Peter MacCallum Department of Oncology, The University of Melbourne, Parkville, VIC Australia; 2 Cancer Immunology Program, Peter MacCallum Cancer Centre, Melbourne, VIC Australia; 3 School of Clinical Sciences, Monash University, Clayton, VIC Australia; 4 Upper Gastrointestinal Translational Research Laboratory, Peter MacCallum Cancer Centre, Melbourne, VIC Australia; 5 Department of Medicine, Royal Melbourne Hospital, The University of Melbourne, Parkville, VIC Australia; 6 Cancer Therapeutics Program, Peter MacCallum Cancer Centre, Melbourne, VIC Australia

## Abstract

The loss of p300/CBP-associated protein (PCAF) expression is associated with poor clinical outcome in gastric cancer, and a potential bio-marker for invasive and aggressive tumors. However, the mechanism linking loss of PCAF to the onset of gastric cancer has not been identified. Given that PCAF and its binding partner transcriptional adaptor protein 3 (ADA3) were recently shown to regulate the intrinsic (mitochondrial) pathway to apoptosis via epigenetic regulation of phosphofurin acidic cluster sorting proteins 1 and 2 (PACS1, PACS2), we analyzed PCAF, ADA3, and PACS1/2 expression in 99 patient-matched surgical samples ranging from normal gastric mucosa, through pre-malignant chronic gastritis and intestinal metaplasia to stage I–III invasive cancers. PCAF mRNA levels were not reduced in either pre-malignant state but were significantly down-regulated in all stages of gastric cancer, commencing at AJCC stage I (*p* < 0.05), thus linking reduced PCAF expression with early malignant change. Furthermore, patients with combined reduction of PCAF and PACS1 had significantly poorer overall survival (*p* = 0.0257), confirmed in an independent dataset of 359 patients (*p* = 5.8 × 10e-6). At the protein level, PCAF, ADA3, and PACS1 expression were all significantly down-regulated in intestinal-type gastric cancer, and correlated with reduced progression free survival. We conclude that a pro-apoptotic mechanism centered on the intrinsic (mitochondrial) pathway and regulated by PCAF/ADA3 can influence the progression from premalignant to malignant change, and thus act as a tumor suppression mechanism in gastric cancer.

## Introduction

The epigenetic modifier p300/CBP-associated factor (PCAF) is a histone acetyl transferase (HAT), the silencing of which is associated with a number of cancers. PCAF is a key component of various heterotypic protein complexes that regulate multiple tumor suppressors and oncogenes, as well as transcription factors that govern cell cycle progression and cell differentiation^1^. For example, PCAF can acetylate p53 in response to DNA damage resulting in cell cycle arrest^2^, or the p65 subunit of NF-κB^3^, an important regulator of genes associated with inflammation and immunity. The significance of PCAF as a tumor suppressor is highlighted by recent studies demonstrating loss of PCAF in esophageal, breast, ovarian, colorectal, and pancreatic cancers^4–8^. In esophageal squamous cell carcinoma, restoring PCAF expression by reversing hypermethylation of the *PCAF* promoter was associated with suppression of tumor cell growth^8^.

PCAF expression is also commonly reduced in stomach cancer^9^, the fifth most frequent malignancy and third commonest cause of cancer deaths, globally^10,11^. Stomach cancer is often detected when the disease is well advanced and curative surgical resection is no longer possible, so that fewer than 20% of patients remain alive 5 years beyond diagnosis. The disease is classified histologically by the Lauren classification into intestinal, diffuse or mixed subtypes^12^. While our understanding of the pathogenesis of gastric cancer remains incomplete^13^, there is compelling evidence that chronic gastritis (CG) arising from *Helicobacter pylori* infection is followed by intestinal metaplasia (IM), a key precursor of malignant change^14^. In their study, Ying and colleagues^9^, found that reduction in PCAF protein expression was associated with reduced disease free interval, although overall survival was unaffected. The authors also showed that reconstituting PCAF expression in an in vitro gastric cancer cell line reduced both its clonogenicity in soft agar and the growth of tumor xenografts in immunosuppressed mice.

We used short hairpin (shRNA) technology to knockdown expression of >1200 candidate genes involved in cell death signaling to identify those that specifically regulate apoptosis activated by the human pro-apoptotic protease granzyme B. This key immune effector protease is secreted by cytotoxic T cells and natural killer cells and brings about target cell death through the mitochondrial pathway^15^. To date, PCAF has been known as a direct regulator of cell growth and proliferation.  However, our screen independently identified PCAF and its binding partner human transcriptional adaptor 3 (ADA3) as proteins whose down-regulation rendered cancer cells refractory to granzyme B delivered by the pore-forming protein perforin^15^. ADA3 is also known to be a critical regulator of DNA repair, and its mislocalization in cells is linked to adverse outcomes in human breast cancer^16^.

Our studies also further elucidated the tumor-suppressing functions of PCAF and ADA3: mechanistically, we showed that enhanced cancer cell survival in response to reduced PCAF or ADA3 was related to reduced expression of phosphofurin acidic cluster sorting proteins 1 and 2 (PACS1, PACS2), as both the *PACS1* and *PACS2* genes are regulated epigenetically by PCAF and ADA3, acting in concert^15,17^. Consequently, knockdown of either PACS protein resulted in survival of cervical cancer and colon cancer cells in vitro, phenocopying the loss of PCAF or ADA3 expression^15,17^. We found that loss of PACS1 or PACS2 protected cells against stimuli that induce mitochondrial outer membrane permeabilisation (MOMP), but through different mechanisms^17^. The PACS protein family regulates protein sorting and trafficking through generic processes including the endosomal transport system^18^, and we showed that disrupting these trafficking pathways suppresses MOMP. In the case of PACS2, this occurs by reducing the activation of the upstream apoptotic mediator BH3 interacting-death domain agonist (BID)^15^, the key substrate cleaved by human granzyme B in the target cell cytosol^19–21^. The reduced generation of activated (truncated) Bid resulted in reduced MOMP, and improved cell survival^15^. By contrast, loss of PACS1 had no effect on Bid trafficking or cleavage; rather, knockdown of PACS1 resulted in disturbed BAX/BAK oligomerization in response to truncated BID and to other BH3-only cell death agonists^17^. Consequently, loss of PACS1 resulted in protection against a broader array of apoptotic stimuli operating through MOMP, including UV radiation and drugs such as staurosporine and etoposide, as well as to granzyme B^17^.

Taken together, the data across our previous studies indicated that PCAF, ADA3, PACS1, and PACS2 together constitute a novel pathway that facilitates cell death through the intrinsic apoptotic pathway, with PCAF and ADA3 playing crucial roles in epigenetically regulating PACS1 or PACS2, which in turn influence MOMP. Given that the previous studies linking PCAF depletion with poor outcomes in stomach and several other forms of cancer^4–9^ did not examine expression of ADA3, PACS1, or PACS2, we decided to determine whether PCAF and/or other components of the apoptotic pathway are down-regulated in primary human gastric cancer and/or in pathologies known to be precursors of malignant transformation.

## Results

### PCAF is down-regulated in early stage stomach cancer, but not in pre-malignant precursor pathologies

A previous study identified a significant reduction in PCAF protein expression in human intestinal-type stomach cancer, however mRNA levels were not reported^9^. To explore this further, we interrogated samples collected from a cohort of 99 patients collected at the time of surgical resection, and which had previously been profiled for their mRNA expression^22^. For some of these patients (*n* = 45), adjacent normal and pre-malignant tissue was also collected and profiled. Histological analysis classified these as normal gastric mucosa (NM), chronic gastritis (CG; *n* = 22), or intestinal metaplasia (IM; *n* = 23). Table 1 lists the characteristics of the entire cohort, in which 48% had early (stage I or II) disease, whereas 51% had advanced (stage III or IV) disease; 52% of the tumors were classified as having intestinal-type morphology and 37% had diffuse morphology.

**Table 1:** Gastric Cancer Cohort characteristics

Characteristic	Count
Gender	
Male	63 (67%)
Female	31 (33%)
Age (years old)	
<60 yo, Mean Age (range)	*N* = 30, 50.7 (32-59)
>60 yo, Mean Age (range)	*N* = 69, 72.78 (60-86)
Mortality	
Alive	36 (36%)
Deceased	63 (64%)
AJCC cancer stage	
I	19 (20%)
II	26 (28%)
III	41 (44%)
IV	7 (7%)
Unspecified	1 (1%)
Tumor site	
Antrum	19 (20%)
Cardia	2 (2%)
Gastro-esophageal junction	18 (19%)
Greater curve-body	26 (28%)
Lesser curve-body	27 (29%)
Body unspecified	1 (1%)
Stomal	1 (1%)
Lauren classification	
Intestinal metaplasia	23 (17%)
Chronic gastritis	22 (16%)
Intestinal	49 (35%)
Diffuse	35 (25%)
Mixed	10 (7%)
Differentiation	
Undifferentiated	26 (28%)
Poor	33 (35%)
Moderate	31 (33%)
Well	3 (3%)
Carcinoma in situ	1
Lymphovascular invasion	
Absent	16 (16%)
Present	36 (36%)
Not determined	47 (47%)

**Fig. 1:**
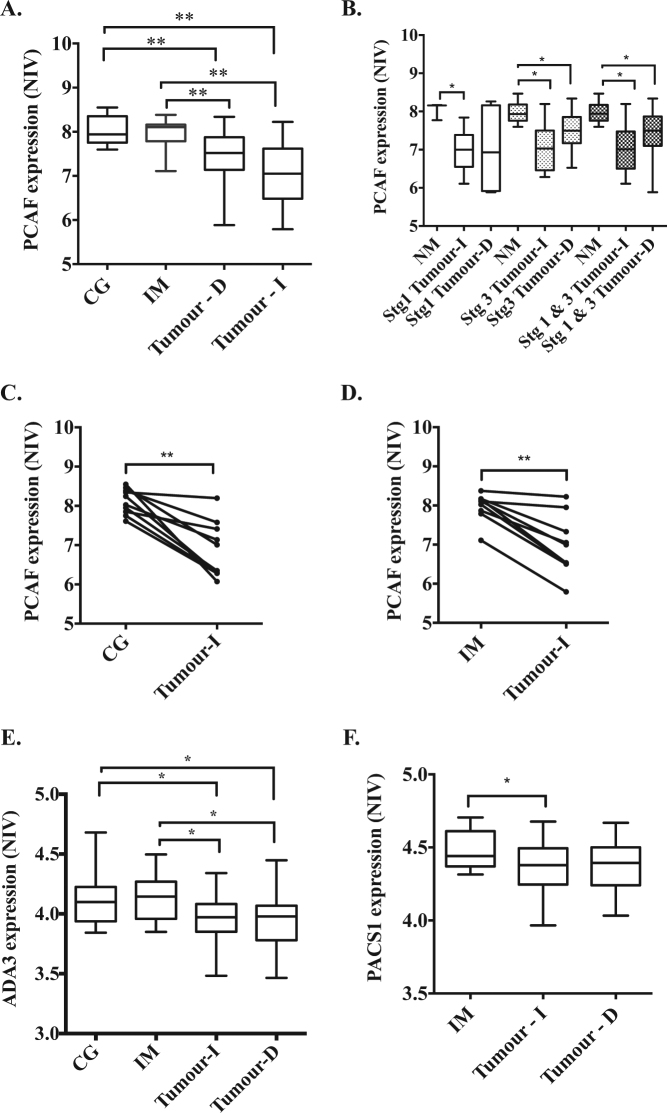
Microarray analysis of gastric tumors reveals reduced PCAF, ADA3, and PACS1 mRNA expression. A cohort of gastric cancer samples and associated premalignant lesions were profiled using Affymetrix U133+2 arrays; data is presented as Normalized Intensity Value (NIV). Tumor tissue was classified histologically into Diffuse (D) or Intestinal (I) subtypes and compared to non-malignant (NM) gastric tissue which was further subdivided into Chronic Gastritis (CG) or Intestinal Metaplasia (IM). **a** shows relative PCAF mRNA expression during the progression to GC; **b** samples were further stratified based on tumor stage and subtype, **c** relative PCAF mRNA expression was then determined for paired CG and intestinal GC and **d** IM and paired intestinal GC samples. The relationship between **e** ADA3 and **f** PACS1 expression during progression was also determined. The boxes on the whisker plots show the mean and 25th percentile values throughout. Error bars are SEM, statistical analysis performed: 2-way ANOVA and Bonferroni test or *t*-test, *P < 0.05 and **P < 0.01

Given that the previous study (9) examined only protein expression, we first quantified the relative PCAF mRNA levels across the cohort. When we pooled the data across the cohort, PCAF expression levels were significantly reduced in both intestinal-type and diffuse cancer tissue, compared to CG and IM (Fig. 1a, *p* < 0.01). When the data were further analyzed to take into account stage of disease, the reduction in PCAF mRNA was evident across all stages for intestinal-type cancers, including the earliest stage, AJCC stage I (Fig. 1b, *p* < 0.05). A similar trend was noted for the diffuse tumors, however the difference only became statistically significant for later stage cancers. Mean PCAF mRNA expression levels were not reduced in stage 3 disease compared with stage I (with both histological subtypes), suggesting that PCAF expression is lost early in the development of gastric cancer, possibly in the transition from pre-malignant IM to invasive cancer (Fig. 1b, *p* < 0.05). We then further compared PCAF expression levels in paired samples from individual patients, and found that mRNA levels were invariably reduced in the cancer, in comparison with premalignant lesions from the same patient (Fig. 1c, d, *p* < 0.01).

We then quantified relative mRNA expression across our cohort for the PCAF binding partner ADA3 and for PACS1 and PACS2, whose genes are epigenetically regulated by PCAF and ADA3^15^. We observed a significant reduction in ADA3 expression in gastric tumor tissue of both intestinal and diffuse types, compared to pre-malignant CG and IM (Fig. 1e, *p* < 0.05). A similar reduction in PACS1 mRNA was observed in intestinal-type cancer tissue compared to IM (Fig. 1f, *p* < 0.05). A similar trend was noted for diffuse cancers but failed to reach statistical significance (Fig. 1f). However, there was no significant difference in PACS2 mRNA levels between cancerous tissue and pre-malignant tissue (data not shown). We were also able to compare mRNA levels for ADA3 and PACS1 by stage of disease, and found that unlike for PCAF, there was no significant association for either antigen with disease stage (data not shown).

**Fig. 2:**
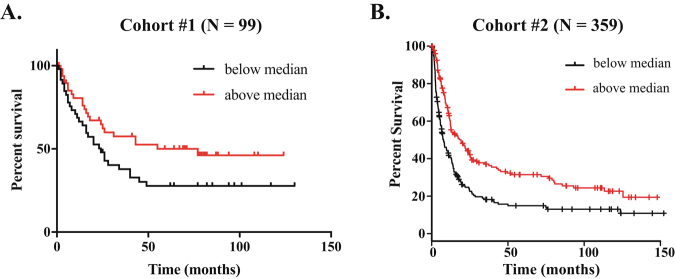
Loss of expression of both PCAF and PACS1 confers reduced overall survival in gastric cancer. Kaplan–Meier curves depicting overall survival of gastric cancer patients with low (below median) or high (above median) expression of both PCAF and PACS1 mRNA. **a** Cohort #1 comprised 99 patients (see reference 22), *p* = 0.0257; **b** Cohort #2 comprised 359 patients (see reference 23), *p* = 5.8 × 10e-6. Statistical analysis was performed using the log-rank test

**Fig. 3:**
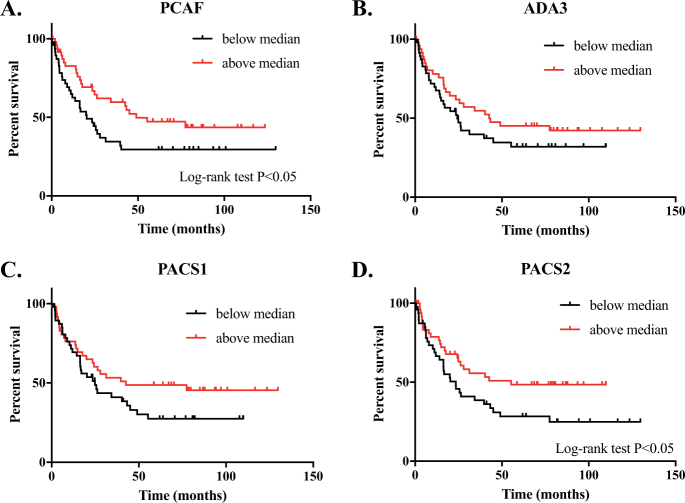
Kaplan–Meier curves depicting progression free survival (PFS) . Expression values were determined for all gastric cancer samples within the cohort. Samples were stratified based on median expression values (low or below median, black; and high or above median, red). Plots compare the progression free survival (PFS) for **a** PCAF (*n* = 50, *p* = 0.04), **b** ADA3 (*n* = 50, *p* = 0.20), **c** PACS1 (*n* = 50, *p* = 0.13), and **d** PACS2 (*n* = 50, *p* = 0.04). Statistical analysis was performed using the log-rank test

We next investigated whether gene expression levels (stratified relative to median expression in our cohort as either “high” or “low”) correlated with clinical outcome. We found no difference in overall survival when the expression levels for PCAF, ADA3, PACS1, or PACS2 mRNA were considered singly (data not shown). However, patients who had reduced expression of both PCAF and PACS1 had significantly reduced overall survival (Fig. 2a, *p* = 0.0257). The observation was validated using a larger, independent dataset comprising 359 gastric cancer patients^23^, confirming the significant role of this pathway in gastric carcinogenesis (Fig. 2b, *p* = 5.8 × 10e-6). There was significantly shorter progression free survival (PFS) for patients with low mRNA expression of either PCAF or PACS2 (Fig. 3a, d, *p* < 0.05). A similar trend was noted for both PACS1 and ADA3 mRNA levels, but neither reached statistical significance (Fig. 3b, c).

### Reduced PCAF, ADA3, and PACS1 protein expression in human gastric tumor tissues

**Fig. 4:**
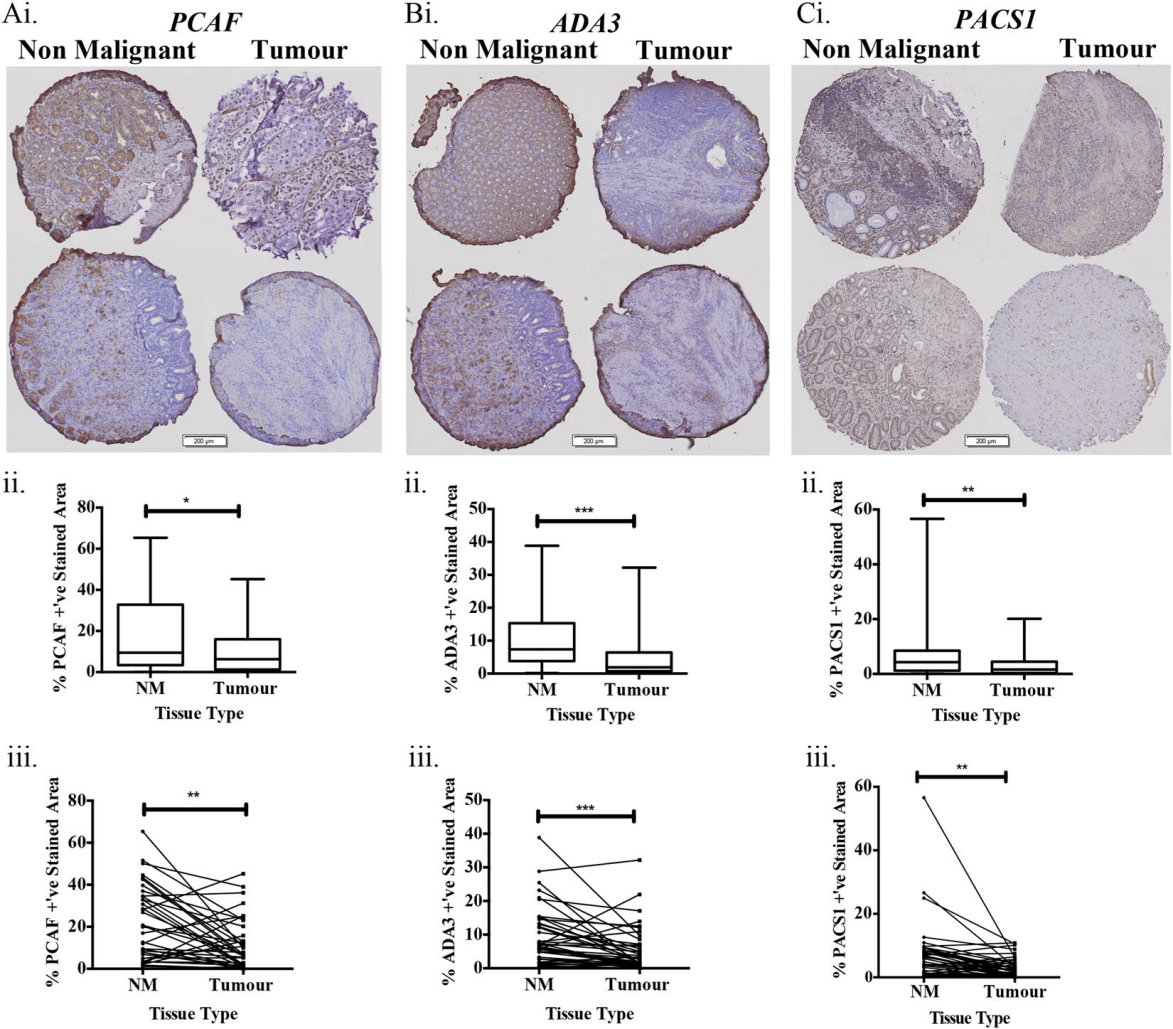
PCAF, ADA3, and PACS1 protein expression is reduced in human gastric tumor tissue. A tissue microarray (TMA) containing gastric tumor tissue (Tumor) and non-malignant gastric tissue (NM), was immunostained for **a** PCAF, **b** ADA3, and **c** PACS1. Data are presented as percentage positive stained area (%+’ve) quantitated by normalizing the relative intensity for each protein to that of the respective control IgG. The process for determining each experimental value in the plots was automated, using MetaMorph® image analysis software (MetaMorph Inc., USA). i Two representative sets of tissue samples for each of the three antigens under study (PCAF, ADA3, PACS1), comparing non-malignant and malignant tumor tissue. ii Whisker plots comparing pooled data across the entire cohort for non-malignant (NM) versus malignant tissue sections. iii Pairwise analysis for TMA %+’ve stained area for gastric tumor tissue to patient matched non-malignant gastric tissue. Error bars are S.E.M., statistical analysis performed: *t*-test, *p < 0.05, **p < 0.01 and ***p < 0.001

Given that the pro-apoptotic proteins PCAF, ADA3, and PACS1 were significantly down regulated in tumor compared to premalignant gastric mucosa at the mRNA level, we examined the expression of the corresponding proteins by immunohistochemistry in a tissue microarray (TMA). The TMA comprised 73 resected stomach cancer specimens and 70 specimens of adjacent non-malignant and premalignant gastric mucosa from the same patients (Table 2). Staining was compared on consecutive tissue sections of the TMA to enable automated (unbiased) image analysis and direct comparison of the samples, with the staining quantified according to the percentage of area stained for each protein under study, in comparison with isotype-matched control IgG. Representative images for each antibody are shown (Fig. 4ai–ci), along with histograms showing the pooled quantified data for each antigen (Fig. 4aii–cii). We found a significant decrease in staining for PCAF, ADA3 and PACS1 in the malignant tissue compared to non-malignant mucosa when the data were pooled across all of the specimens (Fig. 4aii–cii, *p* < 0.05), and an even stronger association when the percentage stained area was compared for matched malignant and adjacent normal mucosa from the same individual (Fig. 4aiii–ciii, *p* < 0.01).

**Table 2:** Tissue microarray gastric cancer cohort tumor characteristics

Tumor characteristics	Count
Gender	
Male	48 (66%)
Female	24 (33%)
Not available	1 (1%)
Age (years old)	
<60 yo, Mean Age (range)	31, 51.87 (43−58)
>60 yo, Mean Age (range)	42, 71.81 (61−86)
Mortality	
Alive	34 (47%)
Deceased	39 (53%)
AJCC	
I	4 (5%)
II	31 (42%)
III	35 (48%)
IV	2 (3%)
Unspecified	1 (1%)
Tumor site	
Antrum	16 (22%)
Cardia	6 (8%)
Gastro-oesophageal junction	6 (8%)
Greater curve-body	19 (26%)
Lesser curve-body	22 (30%)
Body	1 (1%)
Fundus	2 (3%)
Pylorus	1 (1%)
Lauren classification	
Intestinal	34 (47%)
Diffuse	23 (32%)
Mixed	11 (15%)
Not classified	5 (7%)
Differentiation	
Moderate	19 (26%)
Undifferentiated	8 (11%)
Poor	41 (56%)
Well	1 (1%)
Not determined	4 (5%)
Lymphovascular invasion	
Absent	23 (32%)
Present	41 (56%)
Not determined	9 (12%)

## Discussion

Gastric cancer is a deadly disease, a result of asymptomatic early disease leading to late diagnosis and a lack of effective treatment options^24^. The specific mechanisms of tumorigenesis that lead to gastric cancer are incompletely understood. It has previously been observed that the HAT protein PCAF is significantly reduced in human gastric cancer tissue, particularly the intestinal sub-type^9,25^. For this reason, we examined tumor, and where possible, matched normal mucosa and pre-malignant tissues (CG, IM) from a cohort of gastric cancer patients diagnosed with intestinal, diffuse or mixed gastric tumors. We became interested in the clinical observations on PCAF expression because we had independently found from performing a functional genomics (shRNA knock-down) screen that loss of PCAF or its binding partner ADA3 resulted in protection of cancer cell lines from a range of apoptotic stimuli such as anti-cancer drugs, UV irradiation and human granzyme B, that operate by inducing MOMP^15,17^. The studies were carefully controlled and were performed in an isogenic setting; that is, we used either sh or siRNA knock down of individual mRNAs in the same cells and compared the effects to scrambled or irrelevant targeting constructs. We also used immunoblotting and quantitative PCR to ensure that our cellular manipulations had no effect on the expression levels of a broad range of other proteins that regulate MOMP^17^. Our study design was also influenced by more recent advances in our understanding of how PCAF and ADA3 epigenetically co-regulate MOMP to bring about apoptotic cell death, and the involvement of downstream PCAF/ADA3 targets PACS1 and PACS2 in that process^15,17^. Using similar isogenic settings, we showed that the loss of expression of PACS1/2 phenocopied the loss of ADA3/PCAF, which epigenetically regulate their expression.

Along with its binding partner ADA3, PCAF resides in a multi-protein complex that regulates gene transcription^26^. PCAF and ADA3 are critically implicated in cell death mediated through the intrinsic pathway and directly regulate PACS1 and PACS2 expression^15,17^. PACS1 is an adaptor protein that shuttles other proteins between the *trans* Golgi network and endosomes. It typically binds to proteins with acidic cluster motifs and its cargoes thus include a broad variety of proteases, receptors, ion channels and viral proteins, implicating PACS1 as an important factor in maintaining cellular homeostasis^27^. The specific function of PACS1 in apoptosis has not been completely deciphered, but its absence results in the formation of aberrant Bax/Bak multimers and reduced MOMP in cells exposed to a variety of death stimuli^17^. Mutation or deletions of the PACS1 gene locus at chromosome 11q13.1 has been associated with the development of several cancers including cervical cancer^28,29^. Importantly, we have also observed that independently reducing PACS1 expression levels endows significant protection against cell death stimuli that operate through the mitochondrial pathway, phenocopying the loss of PCAF/ADA3^17^. The loss of PACS2 did not qualitatively affect the formation of Bax/Bak oligomers, but exerted a similar effect on MOMP by reducing the processing (or possibly the trafficking) of the BH3-only protein BID, thus damping the death signaling pathways that culminate in Bax/Bak oligomerisation^15^.

In the current study, we observed that PCAF mRNA and protein expression was significantly reduced in all stages of gastric cancer (including the earliest, or AJCC stage 1), but remained at normal levels in adjacent non-malignant or pre-malignant tissues, including in a large series of matched controls. Similar changes were also observed for ADA3 and PACS1. The changes were common to both the intestinal and diffuse forms of the disease: PCAF was significantly reduced in diffuse gastric cancer, PACS1 in the intestinal form, and ADA3 in both subtypes. Overall survival was significantly reduced for patients where the expression of both PACS1 and PCAF were diminished. The latter observation was confirmed in a larger, independent cohort of gastric cancer patients^23^ adding considerable weight to our findings (p value for the larger cohort was 5.8 × 10e-6, as compared with 0.0257 for the original study group) (Fig. 2). Also, progression-free survival was significantly higher in patients where the single mRNAs encoding PCAF (or PACS2, also regulated by PCAF and ADA3) expression was preserved (Fig. 3).

Reduced PCAF, ADA3, and PACS1 expression may have implications for gastric cancer tumorigenesis given our findings that PCAF and ADA3 expression are reduced on stage I disease but not in adjacent tissues affected by pre-malignant change (CG or IM), and that PACS1/PACS2 play an important role as effectors of apoptosis through the intrinsic pathway^15,17^. Our report is the first to link reduced PACS1 levels with gastric cancer. The recently demonstrated pro-apoptotic properties of PACS1 and PACS2 and their transcriptional regulation by ADA3 and PCAF suggests that the four proteins (and possibly others) constitute a novel signaling pathway that acts to suppress the formation of gastric (and possibly some other) cancers. The fact that these changes were not associated with either CG or IM provides early evidence for the hypothesis that defects of this pathway may prompt the change from a pre-malignant state to invasive cancer.

PCAF and ADA3 are two core proteins residing in histone modifying complexes for example in the human ATAC or SAGA complexes and their deregulation has been associated with solid tumor development such as breast, ovarian, colon and esophageal cancers^4,6,8^. These studies further highlight the tumor suppressing functions of PCAF that require further exploration. In healthy cells a dynamic equilibrium state between HATs and histone deacetylases (HDACs) exists that tightly regulates gene transcription. Tumorigenesis can disrupt this equilibrium, resulting in epigenetic reprogramming that affects gene expression enhancing cell survival or proliferation of cancer cells. Current new therapies targeting epigenetic modifications such as selective and non-selective HDAC inhibitors are showing signs of success in hematological malignancies such as leukemia and some solid tumors^30^. However, identifying precisely which epigenetic modifiers are altered in solid tumor models would allow more rational selection of appropriate targeted therapy either as a mono therapy or in combination with conventional agents. For example, one study found that the PCAF genotype that encodes Ser/Ser at codon 386 is found significantly more frequently in hepatocellular cancer^31^, while others showed that the *PCAF* locus is frequently disrupted in esophageal cancers^8,32^. If such mutations were to cause reduced MOMP as shown by us, sensitivity to cancer drugs operating through the mitochondrial pathway might be predicted to improve by adding a Bcl-2 inhibitor to the initial therapy.

This study highlights the potential of using PCAF, ADA3, and PACS1 as biomarkers for early invasive gastric cancer and possibly, to predict response to cancer drugs that activate the mitochondrial cell death pathway. If so, our current study suggests that tracking the expression of mRNA/antigen levels for single proteins may be less informative than in combination, for example PCAF in concert with PACS1, as suggested by this study. Further validation of these markers in other, independent cohorts of gastric cancer patients will be required to establish their significance both in gastric cancer development and as predictors of clinical outcome.

## Materials and methods

### Gastric cancer samples

Gastric tumor (*n* = 99) and matching premalignant samples (chronic gastritis and intestinal metaplasia) were collected from patients undergoing curative resection for GC from Melbourne Australia. Fresh frozen and formalin fixed tissue was collected at the time of surgery, as described previously^33^. RNA was extracted and profiled using Affymetrix U133+2 chips as previously described (GSE51105)^22^. A second, independent sample collection collected from 359 patients^23^ was also interrogated in some analyses, particularly to verify or refute results from our 99-subject study cohort.

### Flow cytometry

For flow cytometry, cells were washed with PBS containing 0.5% FCS then resuspended in Annexin-V buffer with APC Annexin-V (Biolegend, 640919) and 7-AAD-FITC (Beckman Coulter, 559925) and fluorescence was detected on a cytofluorograph.

### RNA extraction and gene profiling

RNA was extracted from cell lines and patient tissue using the RNeasy mini Kit (Qiagen, 74104) according to the manufacturer’s instructions. Primary tissue mRNAs were hybridized to Affymetrix Human Genome U133 plus Genechips HG-U133 Plus 2.0 (Affymetrix, 900470) according to the manufacturer’s instructions, as previously described^22^. The microarray data sets are available at http://www.ncbi.nlm.nih.gov/projects/geo​/ (Accession: GSE1105). Raw data were normalized using RMA with Partek® software as described previously and gene expression values, represented as normalized intensity values, were extracted^33^. Findings were validated using quantitative real time PCR (qPCR) on gastric cancer cell lines. Cell line RNA was used for first strand cDNA synthesis as previously described^15^. HPRT, PACS1, PCAF, and ADA3 genes (primers available on request) were quantified using qPCR, as previously described^15^.

### Tissue microarray and immunohistochemistry

GC and non-malignant tissues were ethanol or formalin fixed, paraffin embedded and used to create tissue microarrays (TMAs). Each tissue array block contained up to 60 samples. Sections of 4 mm were cut from each tissue array block, deparaffinized and dehydrated. For immunohistochemistry, slides were dewaxed and rehydrated using a Leica Auto Stainer (Leica Biosystems, Wetzlar, Germany) followed by high pressure antigen retrieval. Endogenous peroxidase was blocked in 3% H_2_O_2_ washed with PBS 0.05% Tween and blocked in 2.5% normal horse serum. Sections were incubated in normal Rabbit IgG (Santa Cruz, sc-2027), PCAF (Abcam, ab12188), ADA3 (Novus Biologicals, NBP1-90243) or PACS1 (Abnova, PAB23363) overnight at 4 °C. Sections were washed with PBS 0.05% Tween, incubated in ImmPRESS™ anti-rabbit Ig reagent (Vector Laboratories, MP7401) and then washed with PBS 0.05% Tween. Slides were then developed in diaminobenzidine (DAB) peroxidase substrate solution (Vector Laboratories, SK4103), washed in water and counterstained with hematoxylin using a Leica Autostainer (Leica Biosystems, Wetzlar, Germany) and mounted. Slides were scanned using VS120 virtual slide microscope scanner (Olympus, Nagano, Japan) to create images for analysis. The percentage of positive (DAB) stained area for each protein of interest (PCAF, ADA3, or PACS1) was measured using MetaMorph® image analysis software (MetaMorph Inc., TN, USA). A relative area for each tissue sample was quantitated. The percentage staining for each tissue sample was calculated by normalizing the relative staining for each protein to the respective IgG relative DAB stained area. Values were then plotted to create a histogram presented as a mean±SEM or presented as paired data comparing the percentage intensity of non-malignant tissue (NM) to the paired gastric cancer tissue (tumor) samples.

### Statistical analysis

Results are presented as mean ± S.E.M. Statistical differences were evaluated by *t*-test or two-way ANOVA with Bonferroni adjustment by Prism software (GraphPad, CA, USA). A *p*-value of 0.05 was considered significant. Kaplein–Meier curves for progression free survival (PFS) were generated using Graphpad Prism. Samples were divided into those which are low (below median) and high (above median) expressing for each gene of interest. For tissue microarray analysis data was presented in the following groups: gastric tumor tissue for Intestinal (I) or Diffuse (D), non-malignant tissue Chronic Gastritis (CG) or Intestinal Metaplasia (IM).
